# Current Pharmacological Advances in the Treatment of Cardiac Arrest

**DOI:** 10.1155/2012/815857

**Published:** 2011-11-20

**Authors:** Andry Papastylianou, S. Mentzelopoulos

**Affiliations:** Intensive Care Unit, Evagelismos Hospital, 45-47 Ipsilantou Street, Athens 10676, Greece

## Abstract

Cardiac arrest is defined as the sudden cessation of spontaneous ventilation and circulation. Within 15 seconds of cardiac arrest, the patient loses consciousness, electroencephalogram becomes flat after 30 seconds, pupils dilate fully after 60 seconds, and cerebral damage takes place within 90–300 seconds. It is essential to act immediately as irreversible damage can occur in a short time. Cardiopulmonary resuscitation (CPR) is an attempt to restore spontaneous circulation through a broad range of interventions which are early defibrillation, high-quality and uninterrupted chest compressions, advanced airway interventions, and pharmacological interventions. Drugs should be considered only after initial shocks have been delivered (when indicated) and chest compressions and ventilation have been started. During cardiopulmonary resuscitation, no specific drug therapy has been shown to improve survival to hospital discharge after cardiac arrest, and only few drugs have a proven benefit for short-term survival. This paper reviews current pharmacological treatment of cardiac arrest. There are three groups of drugs relevant to the management of cardiac arrest: vasopressors, antiarrhythmics, and other drugs such as sodium bicarbonate, calcium, magnesium, atropine, fibrinolytic drugs, and corticosteroids.

## 1. Introduction

Cardiac arrest constitutes a major health problem with dismal prognosis. Cardiac arrest may be caused either by asystole, pulseless electrical activity (PEA), pulseless ventricular tachycardia (VT), or ventricular fibrillation (VF) [1].

Data from 37 communities in Europe indicate that the annual incidence of Emergency Medical Services- (EMS-) treated, out-of-hospital, cardiopulmonary arrest for all rhythms is 38 per 100,000 population [2]. The annual incidence of EMS-treated VF arrest is 17 per 100,000. Survival to hospital discharge is 10.7% for all-rhythm and 21.2% for VF cardiac arrest. Recent data from 10 North American sites are consistent with these figures: median rate of survival to hospital discharge was 8.4% after EMS-treated cardiac arrest from any rhythm and 22.0% after VF [3].

In-hospital cardiac arrest occurs in 1 to 5 per 1000 patient admissions [4]. The American Heart Association's National Registry of cardiopulmonary resuscitation (CPR) indicate that survival to hospital discharge after in-hospital cardiac arrest is 17.6% (for all rhythms) [5]. The initial rhythm is VF or pulseless-VT in 25% of cases and, of these, 37% leave the hospital alive. After PEA or asystole, 11.5% survive to hospital discharge.

Multistage algorithms have been developed for CPR. In the algorithm for CPR, cardiac arrest rhythms are divided into two groups: shockable rhythms (VF/pulseless VT) and nonshockable rhythms (asystole, PEA). 

The main difference in the treatment of these groups is the need for attempted defibrillation in those patients with VF/pulseless VT. Subsequent actions, including high-quality chest compressions with minimal interruptions, airway management and ventilation, venous access, administration of resuscitation drugs, the identification and correction of reversible factors (hypoxia, hypovolaemia, hypothermia, hypo-/hyperkalaemia (4Hs), thrombosis-coronary or pulmonary, tamponade, toxins, tension pneumothorax (4Ts)), and the use of therapeutic hypothermia in comatose survivors are common to both groups [6]. 

We emphasize the importance of high-quality CPR (including chest compressions of adequate rate and depth, allowing complete chest recoil after each compression, minimizing interruptions in chest compressions and avoiding excessive ventilation), early defibrillation, and the use of therapeutic hypothermia in comatose survivors.

In any case, during CPR, vascular access, drug delivery, and advanced airway management should not cause significant interruptions in chest compressions or delay defibrillation.

In this paper, we summarize current experimental and clinical data on the efficacy and safety of drugs during CPR (vasopressors, antiarrhythmics, and other drugs such as sodium bicarbonate, calcium, magnesium, atropine, fibrinolytic drugs, and corticosteroids).

## 2. Vasopressors

Cardiac arrest is characterized by global ischemia, tissue hypoxia, and acidosis. CPR aims to improve the oxygen supply-demand ratio, in order to reverse tissue hypoxia.

Vasopressors are drugs that have a predominantly vasoconstrictive action on the peripheral vasculature, both arterial and venous. These drugs are used, during cardiac arrest, primarily to increase coronary and cerebral perfusion pressures and flows and thus facilitate ROSC.

Furthermore, during cardiac arrest, there is insufficient release of endogenous vasoconstrictors as part of the stress response, and this is another reason for using vasopressors. To date, there is no placebo-controlled study that shows that the routine use of any vasopressor at any stage during cardiac arrest increases *neurologically intact survival* to hospital discharge. However, there is evidence that vasopressors are associated with an increased rate of ROSC.

Epinephrine and vasopressin are the most commonly studied vasopressors in cardiac arrest. Historically, epinephrine has been used for the treatment of cardiac arrest for over 100 years [7]. However, vasopressin has been the focus of considerable research effort and has gained some popularity as a potential adjunct or alternative to epinephrine. There are no alternative vasopressors (norepinephrine, phenylephrine) with proven survival benefit relative to epinephrine [8, 9].

### 2.1. Epinephrine

**Figure 1:**
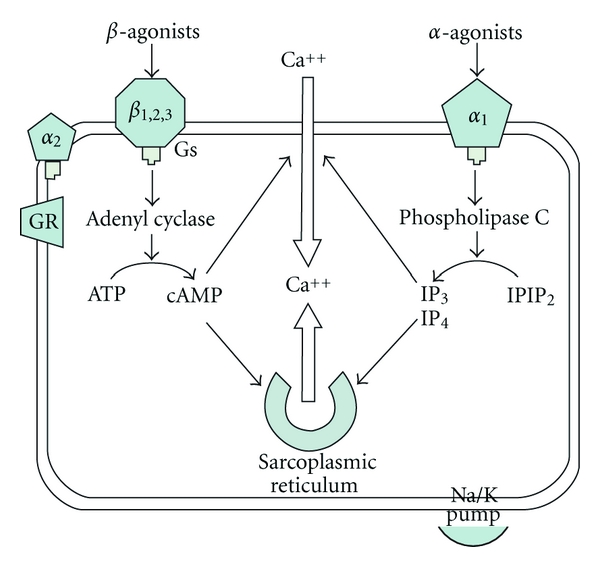
Schematic representation of the action of epinephrine on intracellular calcium in myocytes. ATP: adenosine triphosphate, cAMP: cyclic adenosine monophosphate, Gs: G protein complex, IP: inositol phosphate, PIP_2_: phosphoinositol diphosphate, (adapted from OH'S intensive care manual).

Epinephrine is a powerful agonist at both *α*- and *β*-Adrenergic Receptors. Stimulation of the *β*-receptor activates Gs-proteins, which in turn activate adenyl cyclase and thus lead to the generation of circular adenosine monophosphate (cAMP). In cardiac myocytes, this leads to increased intracellular Ca^2+^ concentration and contractility (inotropic effect). The stimulation of the *β*-receptor can also cause positive chronotropic effects (increased heart rate) and dromotropic effects (augmented conduction). In contrast, *α*1-receptor stimulation results in the activation of phospholipase C, increased formation of inositol phosphates (IP_3_, IP_4_) and diacyl glycerol, and smooth muscle contraction (Figure 1).

Epinephrine has been used for the treatment of cardiac arrest mostly for its *α*-adrenergic effects which cause systemic vasoconstriction, and increase coronary and cerebral perfusion pressures. The beta-adrenergic actions of adrenaline (inotropic, chronotropic) may increase coronary and cerebral blood flow, but may also have catastrophic effects such as the increase of myocardial oxygen consumption, ventricular arrhythmias (particularly when the cardiac tissue is acidotic), transient hypoxemia secondary to attenuation of hypoxic pulmonary vasoconstriction, and consequent increase in intrapulmonary, arteriovenous shunting, impaired microcirculation [10], and heart failure after ROSC [11]. Despite these deleterious effects and the limited evidence of benefit from the use of epinephrine during cardiac arrest, the improved short-term survival documented in some studies [12, 13] supports its continued use in cardiac arrest. 

There are few data on the pharmacokinetics of epinephrine during CPR. In animal studies, peak plasma concentrations occur at about 90 seconds after a peripheral injection. The optimal dose of epinephrine is not known. 

A meta-analysis of studies comparing standard- (1 mg or 0.02 mg/kg) versus high dose (5–15 mg) epinephrine (3199 patients received high-dose and 3140 patients received standard dose), demonstrated improved ROSC in the high dose epinephrine group but failed to demonstrate any long-term survival benefit of the high and/or escalating dose of epinephrine [14]. Considering these results, Babbs et al. [15] concluded that a high initial dose of epinephrine in cardiac arrest may increase coronary perfusion pressures** (**CPP) and ROSC, but may also exacerbate postresuscitation myocardial dysfunction and cause harm. They concluded that high doses of epinephrine do not improve long-term survival and neurological outcome. Laboratory studies also documented adverse effects of high-dose epinephrine when given during CPR, including postresuscitation hyperadrenergic state [16], myocardial necrosis [17], worsened postarrest cardiomyopathy [18], and greater early mortality relative to the standard dose [19].

In view of the above, current guidelines (2010) for ALS recommend a standard dose of 1 mg of epinephrine (IV/IO) every 3 to 5 minutes during adult cardiac arrest (Class IIb, LOE A). Higher doses may be indicated to treat specific problems, such as a beta-blocker or calcium channel blocker overdose [6]. 

If the initial arrest rhythm is PEA or asystole, the administration of epinephrine is recommended as soon as vascular access is obtained. For VF or pulseless VT, the administration of epinephrine is recommended, after the third shock, and upon the resumption of chest compressions [20]. 

### 2.2. Vasopressin

The potentially catastrophic beta-effects of epinephrine led to the exploration of alternative vasopressors. In survivors of cardiac arrest endogenous vasopressin levels are higher than in nonsurvivors [21]. 

Arginine vasopressin (AVP)—a hypothalamic hormone—also known as antidiuretic hormone is a nonapeptide, that is, a 9 amino acid peptide. AVP is released from the posterior pituitary mostly when plasma osmolality is increased or plasma volume is reduced.

It acts via specific G-protein-coupled receptors. Three specific vasopressin receptors (V_1,2,3_) are responsible for vasopressin's pharmacological effects. V_1_ receptors are located in vascular smooth muscle and mediate vasoconstriction, V_2_ receptors are located in the distal convoluted tubules and medullary collecting ducts and mediate antidiuresis, V_3_ receptors are located in the anterior hypophysis and pancreatic isles and seem to affect insulin secretion, facilitate adrenocorticotropin release, and modulate memory, body temperature, and blood pressure.

Vasopressin is metabolized by vasopressinases in the liver and kidney and its half-life is 10–35 min. It has several advantages over epinephrine in CPR because it improves cerebral and myocardial blood flow via V_1_-receptor mediated vasoconstriction, without the unwanted beta-effects of epinephrine. Also, in contrast to epinephrine, vasopressin's vasoconstrictive effect is preserved during hypoxia and severe acidosis. In addition, the risk of postresuscitation myocardial dysfunction is lower [22]. 

The effect of vasopressin during CPR has been extensively investigated in laboratory models. Animal studies demonstrated that vasopressin versus epinephrine increased blood flow in vital organs [23], improved cerebral oxygen delivery [24], and increased the probability of restoring spontaneous circulation [25] and neurologic outcome [26], as compared with epinephrine. The combination of vasopressin and epinephrine tripled coronary perfusion pressure versus either epinephrine or vasopressin alone, in a pig model of prolonged asphyxia [27]. In contrast to the animal studies, human trials showed no clear benefit of vasopressin over epinephrine in the treatment of cardiac arrest. 

Three randomised controlled trials [28–30] and a meta-analysis [31] demonstrated no difference in outcomes (ROSC, survival to hospital discharge, or neurological outcome) between vasopressin and epinephrine used as first line vasopressor in cardiac arrest. Similarly, there was no evidence of harm from the use of vasopressin given during CPR. 

Lindner et al. [28] conducted a randomized controlled trial involving 40 patients with out-of-hospital VF that was unresponsive to defibrillation. Vasopressin versus epinephrine increased 24-hour survival, but there was no between-group difference in the other outcomes (i.e., ROSC, survival on hospital admission, survival to hospital discharge, and neurological outcome).

Stiell et al. [29] conducted a randomized controlled trial involving 200 patients with in-hospital cardiac arrest and showed no statistically significant differences in outcomes (survival to 1 hour, survival to hospital discharge and neurological outcome) between vasopressin and epinephrine.

Wenzel et al. [30] conducted a large, multicenter, randomized, controlled study involving 1219 patients with out-of-hospital cardiac arrest and showed no overall statistically significant differences in outcomes (ROSC, survival to hospital discharge, and neurological outcome) between the vasopressin and epinephrine groups. Subgroup analyses showed that the use of vasopressin in patients with asystole was associated with higher rates of hospital admission and survival to hospital discharge.

Two more recent, randomized, controlled trials [32, 33] showed no difference in outcomes (ROSC, survival to hospital discharge, and neurological outcome) when comparing epinephrine in combination with vasopressin versus epinephrine alone in cardiac arrest. In the large clinical trial in France, a total of 1442 patients with out-of-hospital cardiac arrest received a combination of epinephrine and vasopressin, and 1452 received epinephrine alone. The authors concluded that compared with epinephrine alone, the combination of vasopressin and epinephrine during ALS for out-of-hospital cardiac arrest does not improve outcome [33].

A recent study reported that the combination of vasopressin and epinephrine during cardiopulmonary resuscitation increased end-tidal carbon dioxide and mean arterial blood pressure [34]. This improvement in these surrogate measures of vital-organ perfusion may have been the mechanism responsible for the subsequent, observed increase in short-term survival.

Mentzelopoulos et al. [35] conducted a single-center, prospective, randomized, double-blind, placebo-controlled trial, involving 100 patients with in hospital refractory cardiac arrest and studied the efficacy of combined vasopressin-epinephrine during CPR with corticosteroid supplementation during and after CPR, compared with epinephrine alone, without corticosteroid supplementation. The authors concluded that compared with epinephrine alone, the combination of vasopressin and epinephrine with corticosteroid supplementation during and after CPR improves ROSC and survival to hospital discharge.

In view of the above, the American Heart Association current guidelines (2010) for ALS recommend that vasopressin (40 units) may replace either the first or the second dose of epinephrine in the treatment of cardiac arrest (Class IIb, LOE A).

## 3. Antiarrhythmics

Antiarrhythmic drugs produce pharmacologic effects by blocking sodium, potassium, and calcium ion channels present in the heart. The cardiac action potential is the result of multiple inward and outward ion currents with specific ion channels responsible for each of its five phases. The effects of antiarrhythmic drugs on the action potential and effective refractory period of the cardiac action potential determine the clinical effect of these drugs. 

Lidocaine has traditionally been the antiarrhythmic drug of choice for the treatment of shock-resistant VF and for the prevention of VF recurrence after out of hospital cardiac arrest [36]. However, amiodarone has been shown to increase survival to hospital admission after out of hospital cardiac arrest when compared with placebo or lidocaine and is now recommended as the antiarrhythmic drug of choice for the treatment of refractory VF/VT. Lidocaine is indicated in refractory VF/VT when amiodarone is unavailable.

### 3.1. Amiodarone

Amiodarone is a potent antiarrhythmic agent with a complex electrophysiological and pharmacological profile. It is primarily a Vaughan Williams Class III agent; it acts by inhibiting the inward potassium current. It also blocks sodium and calcium channels and has antiadrenergic effects (noncompetitive blockade of alpha and beta receptors). It prolongs the duration of the action potential, increases the refractoriness of all cardiac tissue, and also prolongs the QT interval. Amiodarone is effective in suppressing both supraventricular and ventricular tachyarrhythmias. 

Amiodarone exerts important cardiovascular effects. It dilates coronary arteries and increases coronary blood supply, and it causes peripheral arterial vasodilation and decreases systemic vascular resistance. Hypotension and bradycardia are the major acute adverse effects from amiodarone. They are related to the rate of infusion and to the solvent (polysorbate 80 and benzyl alcohol) which causes histamine release [37]. These adverse effects can be prevented by slowing the rate of infusion and can be treated with fluids and/or inotropic drugs. Recently, a new aqueous formulation of intravenous amiodarone, that is, relatively free from these adverse effects was approved for use in the United States [38]. An animal study on refractory VF noted that the combination of amiodarone and epinephrine administered together produced haemodynamics as good as epinephrine alone [39]. 

In two randomized, double-blind clinical trials, amiodarone has been shown to be superior to both placebo and lidocaine in improving survival to hospital admission for patients with out-of-hospital refractory VF/pulseless VT [40, 41]. 

The ARREST trial involved 504 patients randomized to receive either amiodarone (300 mg bolus after the failure of three shocks and administration of epinephrine) or placebo; 44% of amiodarone-treated patients and 34% of placebo-treated patients survived to hospital admission (*P* = 0.03) [40]. 

The ALIVE trial involved 347 patients randomized to either amiodarone (initial bolus dose of 5 mg/Kg followed, if needed, by a second dose of 2.5 mg/Kg) or lidocaine (initial bolus dose of 1.5 mg/Kg, repeated once, if needed); more patients treated with amiodarone survived to hospital admission, compared with patients treated with lidocaine (23% versus 12%, *P* < 0.005) [41]. 

In view of the above, the American Heart Association current guidelines (2010) for ALS recommend that amiodarone may be considered for refractory VF/pulseless VT (Class IIb, LOE B), with an initial dose of 300 mg, which can be followed by one dose of 150 mg.

### 3.2. Lidocaine

Lidocaine, long considered an important antiarrhythmic drug for refractory VF/pulseless VT, is now relegated to a second-choice option. It decreases ventricular automaticity. It also has a sodium channel-blocking effect, which is increased in myocardial ischaemia. Signs of lidocaine toxicity such as paraesthesia, confusion, and convulsions may occur in a dose-related manner. It is considered that a safe dose of lidocaine must not exceed 3 mg/Kg over the first hour of administration.

In refractory VF/pulseless VT, lidocaine may be considered if amiodarone is not available (Class IIb, LOE B). The initial dose is 100 mg (1–1.5 mg/Kg) with additional bolus(es) of 50 mg up to the maximum dose of 3 mg/Kg.

### 3.3. Magnesium

Magnesium is a cofactor for many enzyme systems, including the myocardial sodium/potassium ATPase. It is an emerging antiarrhythmic agent that may be best classified as a sodium/potassium pump agonist. Magnesium has many electrophysiological effects, including blocking atrial L and T type calcium channels. It prolongs both atrial refractory period and conduction, inhibits potassium entry, and suppresses ventricular after-depolarizations.

The electrophysiological effects of magnesium are more potent in the presence of increased extracellular potassium, and thus the usefulness of magnesium seems to be greater in the presence of ischemia, where loss of potassium from the cell is a major consequence.

Intravenous magnesium sulphate is indicated in torsades de pointes associated with prolonged QT interval, ventricular, or supraventricular tachycardia associated with hypomagnesaemia, and digoxin toxicity.

In two studies, magnesium sulphate has been shown to facilitate termination of torsades de pointes associated with a prolonged QT interval [42, 43]. A bolus dose of 1 to 2 g of magnesium sulphate diluted in 10 mL of 5% dextrose in water is recommended (Class IIb, LOE C).

It was assumed that magnesium might exert beneficial effects in cardiac arrest, mainly due to its antiarrhythmic and calcium-channel blocking properties [44]. However, three randomised controlled trials [45–47], have failed to demonstrate any benefit of giving magnesium routinely during cardiac arrest.

In view of the above, the American Heart Association guidelines for ALS (2010) do not recommend the routine administration of magnesium sulfate in cardiac arrest (Class III, LOE A), unless torsades de pointes is present.

## 4. Other Drugs

There is no evidence that the routine administration of other drugs such as atropine, calcium, sodium bicarbonate, or fibrinolytic drugs during cardiac arrest increases survival to hospital discharge. The administration of these drugs may be considered in specific cases of cardiac arrest.

### 4.1. Atropine

Atropine is an anticholinergic drug, an ester of tropic acid and tropine. It blocks muscarinic acetylcholine receptors, and, thus, it blocks the effect of the vagus nerve on both the sinoatrial node and the atrioventricular node, and thus increases sinus node automaticity and facilitates atrioventricular conduction.

Atropine is administered in doses of 0.6–3.0 mg IV to counteract bradycardia in the presence of hypotension and to prevent the bradycardia associated with vagal stimulation. Side effects of atropine are dose-related. A dose of ≤0.5 mg may produce an agonist action with a resulting bradycardia. The side effects of atropine are delirium, tachycardia, increase in cardiac work and ventricular arrhythmias, coma, hot skin, blurred vision, and/or urinary retention, pupillary muscle dilation.

The effect of atropine during CPR has been questioned.

In 1979, Brown et al. [48] noted the possible role of the parasympathetic nervous system in cardiac arrest and reported that atropine is beneficial during cardiac arrest. Due to this report, the AHA included atropine in the treatment of asystole.

In 1981, Coon et al. [49] failed to demonstrate any benefit from the administration of atropine in out-of-hospital asystole or PEA.

Stueven and colleagues [50] reported that patients who received atropine had better resuscitation rates and better survival when atropine was given late during ALS. Considering that there was no record of the rhythm at this point, we cannot exclude the possibility that patients had begun to regain a pulse when atropine was given. Recent studies have failed to show any benefit from the use of atropine in out of hospital or in hospital cardiac arrest [51–53].

Atropine is no longer included in the ALS algorithm.

### 4.2. Calcium

Calcium ions are involved in cellular excitation, excitation-contraction coupling, and muscle contraction in cardiac, skeletal, and smooth muscle cells. Increased extracellular calcium increases intracellular calcium concentrations, and the force of contraction of cardiac myocytes and vascular smooth muscle cells. 

There is a direct correlation between duration of resuscitation, low serum ionized calcium levels and mortality. However, high plasma calcium concentration after exogenous calcium injection may promote myocardial damage and impair neurological recovery. Improved survival was suggested by case reports of calcium use in children during cardiac surgery [54]. However, studies of calcium during cardiac arrest failed to demonstrate any beneficial effect on survival [55–57].

Calcium is not recommended for the treatment of cardiac arrest (Class III, LOE B). Administration of calcium during resuscitation is considered only in specific circumstances such as hyperkalaemia, hypocalcaemia, and overdose of calcium channel-blocking drugs.

### 4.3. Sodium Bicarbonate

During cardiac arrest and CPR, combined respiratory and metabolic acidosis arises from the carbon dioxide retention as pulmonary gas exchange ceases and from the reduction in cellular oxygen availability which leads to the development of anaerobic metabolism with lactic acidosis. Severe acidosis inhibits myocardial contractility and also reduces the responsiveness to catecholamines. 

Before 1986, sodium bicarbonate was routinely used during CPR, even without knowledge of the patient's acid-base status. This practice was changed due to the potential adverse effects of buffer therapy and due to the clinical studies that failed to demonstrate any advantage of using sodium bicarbonate during cardiac arrest [58–60]. 

Buffer therapy during CPR can exacerbate intracellular acidosis since it generates carbon dioxide that freely diffuses across cellular membranes. It is estimated that 1 mEq/kg of sodium bicarbonate, given intravenously, produces approximately 180 mL of carbon dioxide, requiring a transient doubling of alveolar ventilation to prevent hypercarbia. Sodium bicarbonate during CPR may also cause hypernatraemia and hyperosmolality to an already compromised circulation and brain, metabolic alkalosis and a leftward shift in the oxygen haemoglobin saturation curve, which further inhibits the release of oxygen to the tissues. Mild acidaemia causes vasodilation and thus increases cerebral blood flow. 

The routine administration of sodium bicarbonate is not recommended in patients with cardiac arrest (Class III, LOE B). Sodium bicarbonate may be given in cases of tricyclic antidepressant overdose and hyperkalaemia.

### 4.4. Fibrinolytic Drugs

Thrombolytic therapy during CPR has two major effects. First, it can be effective in acute myocardial infarction (AMI) or massive pulmonary embolism (PE), which are common causes of cardiac arrest. Second, it may improve microcirculatory reperfusion after ROSC. Cardiac arrest and CPR are associated with a marked activation of coagulation without adequate fibrinolysis [61]. The formation of microthrombi impairs microcirculation (“no-reflow” phenomenon) and contributes to cerebral dysfunction. Thrombolytic therapy can dissolve intravascular blood clots and improve cerebral microcirculation [62]. 

CPR had been considered in the past as a relative contraindication for thrombolysis because of the potential risk for bleeding complications in association with chest compressions. However, currently available data do not confirm the aforementioned speculation [63], and ongoing CPR is not a contraindication to thrombolysis.

Case reports where thrombolysis was used as successful “last resort” therapy (i.e., when the sole alternative would be CPR termination [64]) were followed by several studies suggesting that thrombolysis during CPR benefits patients with pulmonary embolism, or acute myocardial infarction, or those who had been unresponsive to conventional resuscitation efforts [65–72]. 

Conversely, two large clinical trials failed to show any benefit in outcome with fibrinolytic therapy during CPR [73, 74]. 

Abu-Laban et al. [73] conducted a randomized, double-blind, placebo-controlled trial on out-of-hospital thrombolysis during cardiac arrest. Study participants had PEA, and in more than one-third of them the collapse was unwitnessed. The authors found no evidence of thrombolysis benefit. The study was criticized mainly for its limited statistical power.

A multicenter European study [74] investigated whether thrombolysis with the use of tenecteplase during CPR improves survival in witnessed out-of-hospital cardiac arrest of presumed cardiac origin. The results showed no improvement in survival. Despite these negative results, the authors argued that their findings do not suggest that thrombolysis should be withheld in patients with cardiac arrest, if the primary pathologic condition is known to be responsive to such treatment.

Thrombolytic therapy should not be routinely used in cardiac arrest (Class III, LOE B). The administration of thrombolytic therapy may be considered on an “empirical basis,” when pulmonary embolism is presumed or known to be the cause of cardiac arrest (Class IIa, LOE B). In such cases, CPR should be performed for at least 60–90 min before ending the resuscitation attempts [63, 75].

### 4.5. Corticosteroids

Relative to other stress states, cardiac arrest is associated with lower cortisol levels during and after CPR [76, 77]. This adrenocortical dysfunction results in hypotension and shock.

“Postresuscitation disease,” first described by Negovsky [78], shares common features with sepsis such as reversible myocardial dysfunction, vasodilatation, coagulopathy, and plasma cytokine elevation [76]. 

Glucocorticoids modulate vascular reactivity to catecholamines [79, 80] and decrease the production of vasodilators such as nitric oxide [81]. 

Most studies suggest that serum cortisol levels are higher in survivors of cardiac arrest [82] and low serum cortisol levels are associated with early postresuscitation mortality [83, 84].

In a prospective, nonrandomized, open-labeled clinical trial, patients receiving hydrocortisone during resuscitation had a significantly higher ROSC rate than those receiving placebo. In this study there was no significant difference between the two groups in terms of short-term survival and hospital discharge [85]. 

In a prospective, randomized, double-blind, placebo-controlled trial, the combination of vasopressin-epinephrine-methylprednisolone during CPR followed by hydrocortisone, when post-ROSC shock was present, was compared to epinephrine and placebo. The authors concluded that combination therapy increased ROSC and survival to hospital discharge [35]. 

Further studies are needed to investigate the effect of corticosteroid supplementation during and after CPR in order to cover some knowledge gaps in this field.

## 5. Conclusion

For CPR, we emphasize the importance of high-quality chest compressions (rate, depth, recoil), with minimum interruptions and early defibrillation when appropriate. In any case, drug delivery should not cause significant interruptions in these interventions.

No drug has been definitively shown to improve survival to hospital discharge after cardiac arrest. However, evaluation of drugs is difficult after prolonged ischemia time, which anyway minimizes the probability of survival. Future studies might benefit from applying an upper limit for the time from collapse to the administration of drugs (e.g., 5–10 min), in order to avoid the inclusion of potentially “futile cases.”

Adrenaline remains the drug of choice during cardiac resuscitation and other drugs such as atropine, sodium bicarbonate, calcium, magnesium and fibrinolytic drugs may be considered only in specific circumstances.

Corticosteroids during and after CPR seem to confer benefits with respect to hemodynamics, intensity of post-resuscitation systemic inflammatory response and organ dysfunction.

Further studies are needed to investigate the effect of corticosteroid supplementation during and after CPR and the effect of the combination of adrenaline and vasopressin during cardiac resuscitation in order to cover some knowledge gaps in the field.
